# Methodological Considerations in Saliva‐Based Biomarker Research: Addressing Patient‐Specific Variability in Translational Research Protocols

**DOI:** 10.1002/cpz1.70235

**Published:** 2025-10-28

**Authors:** Shirleen Xu, Abdul Nabiel Mumuni, Ralph Thadeus S. Tuason, Katherine A. Maki

**Affiliations:** ^1^ Translational Biobehavioral and Health Promotion Branch, Clinical Center National Institutes of Health Bethesda Maryland

**Keywords:** oral health, periodontal diseases, saliva, salivary biomarkers, smoking

## Abstract

Saliva plays a central role in maintaining oral homeostasis by supporting tooth integrity, providing lubrication, and functioning as an antimicrobial wash. It also serves as a transport medium, carrying byproducts and signaling metabolites across oral niches and through the gastrointestinal tract. Because of its biological relevance and ease of collection, saliva is increasingly used as a noninvasive biospecimen for measuring cortisol, cytokines, and metabolites. However, the validity and reliability of saliva as an indicator of local and systemic biomarkers remain under investigation across diverse populations and research applications. Specific patient populations (e.g., individuals with alcohol use disorder) are particularly vulnerable to oral health problems, periodontal disease, and high rates of nicotine use. In addition to behavioral factors (e.g., food, drink, toothbrushing, and mouthwash), patient‐specific variables can introduce contaminants such as nicotine and blood into saliva, potentially compromising the accurate measurement of analytes of interest. Protocols that account for possible contaminants are essential to ensure rigorous and reproducible biomarker research. Assessing factors such as pH, flow rate, and visible discoloration helps reduce limitations in analysis and improves interpretation in studies that include heterogeneous populations and health behaviors. Yet, the literature provides limited guidance on standardized methods for saliva collection, processing, and measurement of patient‐specific confounders alongside analytes of interest. This protocol addresses these gaps by presenting detailed methodologies for saliva collection and processing, assessment of quantitative and qualitative salivary properties, and quantification of patient‐specific modifiers. These approaches support reproducible diagnostics and have applications in populations with high rates of smoking, periodontal disease, and alcohol use. Published 2025. This article is a U.S. Government work and is in the public domain in the USA. Current Protocols published by Wiley Periodicals LLC.

**Basic Protocol 1**: Saliva collection by the passive drool method

**Basic Protocol 2**: Processing, storage, and characterization of saliva (visual assessment scale, pH, and flow rate)

**Basic Protocol 3**: Quantification of cotinine in salivary supernatant

**Basic Protocol 4**: Quantification of transferrin in salivary supernatant

## Introduction

Saliva is a complex and dynamic biofluid that plays essential roles in maintaining oral health. Through its flow and composition, saliva supports tooth integrity via remineralization, provides lubrication to protect oral tissues, and serves as a first line of defense against pathogens through its antimicrobial properties (Pedersen et al., 2018b; Tabak, 2006). Beyond these local functions, saliva also serves as a conduit for systemic communication. Salivary secretions transport microbial byproducts, host metabolites, hormones, and inflammatory mediators across the oral cavity and into the gastrointestinal tract, contributing to digestion, immune defense, and whole‐body homeostasis (Maki et al., 2021; Pedersen et al., 2018a; Schmidt et al., 2019). The oral microbiome (i.e., the bacterial communities that live in the mouth) is shaped by environmental exposures and host factors, and interacts with salivary elements to influence inflammatory signaling, energy metabolism, and immune regulation (Baker et al., 2024). Together with these microbiome‐driven effects, saliva's broader functions position it as a key mediator and indicator of oral and systemic health, underscoring its value as a biospecimen for biomedical research.

The accessible and noninvasive nature of saliva collection presents unique advantages compared to blood or tissue sampling. Saliva can be obtained repeatedly with minimal burden to participants, making it especially well‐suited for translational and clinical research where longitudinal data are critical. Consequently, salivary bioscience has been applied to a wide range of contexts, including disease diagnostics, drug level quantification, and measurement of biomarkers including stress hormones, inflammatory cytokines, and immunoglobulins (Fuentes et al., 2014; Granger et al., 2012b; Tabak, 2007). These applications have extended to diverse populations, from pediatric cohorts to older adults, and in fields spanning psychiatry, endocrinology, immunology, and oral health research (Granger et al., 2012a; Hamilton et al., 2022; Segal, 2016).

Despite its promise, the use of saliva in biomarker research is complicated by methodological challenges and biological heterogeneity. Many analytes occur in lower concentrations in saliva than in blood, necessitating sensitive assays and rigorous processing protocols. In addition, salivary measures are shaped by both systemic physiology and local oral conditions, complicating interpretation. Patient‐specific and clinical factors such as nicotine use, periodontal disease, and oral bleeding can alter salivary composition and introduce contaminants that compromise analyte quantification and reproducibility when not adequately addressed (Benowitz, 1983; Kang & Kho, 2019; Kivlighan et al., 2004). Broader influences such as diet, stress exposure, adverse childhood experiences, medications, and comorbid health conditions can further contribute to variability in salivary biomarkers (Riis et al., 2021). A lack of standardized collection and analytic approaches compound these challenges, limiting the reliability of saliva as a diagnostic fluid. As the field of salivary bioscience evolves, many of these sources of variability can be anticipated, measured, and controlled through careful study design and standardized protocols.

Individuals with alcohol use disorder (AUD) exemplify a population with distinct oral health challenges that can influence the interpretation of salivary biomarkers without proper study planning (Barb et al., 2022). Heavy alcohol use is linked to dental caries, periodontal disease, edentulism, and mucosal lesions, driven by reduced hydration, decreased salivary flow, and the high sugar content of many alcoholic beverages (Çetinkaya & Romaniuk, 2020; Genco & Borgnakke, 2013). These effects are compounded by malnutrition, food insecurity, and limited preventative dental care, further elevating risk for oral disease (Poudel et al., 2023; Shoff et al., 2025). The previously mentioned patient‐specific and clinical factors that influence salivary biomarkers (e.g., blood contamination and heavy nicotine exposure) introduce confounders that further complicate measurement and interpretation of salivary biomarkers (Benowitz, 1983; Kang & Kho, 2019; Kivlighan et al., 2004). These challenges underscore the need for tailored protocols that address physiological and behavioral characteristics encountered when using salivary bioscience to study specific populations, such as patients with AUD.

Other conditions or populations with intersecting oral health and systemic conditions present their own unique considerations for salivary biomarker research. Patients with metabolic disorders such as diabetes or obesity experience systemic inflammation and dysregulated immune responses that extend to the oral cavity, altering salivary composition. Autoimmune diseases, including Sjögren's syndrome and rheumatoid arthritis, are characterized by salivary gland dysfunction and xerostomia, reducing flow rates and changing protein profiles (Proctor & Shaalan, 2021; Zhou & Liu, 2023). Psychosocial factors such as chronic and current perceived stress, depression, and exposure to early‐life adversity are also linked to alterations in oral health and salivary biomarkers, underscoring the need to integrate behavioral context into protocol design (An et al., 2015; de Mendonça Filho et al., 2023; Ford et al., 2020). Exposure to early‐life adversity is of particular concern because it has been shown to manifest in the form of oral pathology (Bahanan & Ayoub, 2023) and can increase the risk of developing psychological and physical health problems, including AUD and increased risk for cardiovascular disease (Duffy et al., 2018; Kirsch et al., 2025; Maki et al., 2025). These risks may be related to disruptions of the hypothalamic‐pituitary‐adrenal axis and immune function during critical developmental periods, although the mechanisms are not completely understood (Koss & Gunnar, 2018). Although early‐life adversity has unique developmental consequences, adverse experiences at any life stage can affect stress physiology, oral health, and salivary biomarkers, with the timing of exposure influencing the severity and persistence of these effects. Pediatric and geriatric populations may present additional complexities due to developmental and age‐related changes in oral physiology, immune function, and health behaviors (Kudielka et al., 2009; Shaw et al., 2013; Wiener et al., 2010). Collectively, these examples illustrate the wide relevance of standardized, context‐aware salivary protocols for translational research.

The three complementary protocols presented here are designed to address these challenges in salivary bioscience and generate reliable and interpretable data by optimizing biospecimen handling while controlling for patient‐specific factors in translational research studies. Data obtained from these methods include quantitative and qualitative indices of saliva (including pH, flow rate, and blood contamination or discoloration) as well as contextual modifiers (such as nicotine exposure and indicators of periodontal disease). These multimodal data provide a framework to interpret salivary biomarkers both as reflections of oral health and homeostasis and as potential indicators of systemic physiology. By explicitly accounting for these challenges, the methods described here provide investigators with practical solutions for enhancing rigor and reproducibility in salivary bioscience. Basic Protocol 1 outlines standardized procedures for saliva collection, initial processing, and storage. Basic Protocol 2 describes approaches for measuring and recording quantitative and qualitative salivary properties, including pH, flow rate, and visible blood contamination. Basic Protocols 3 and 4 present procedures for evaluating patient‐specific oral health modifiers, such as nicotine exposure and periodontal indicators, which can impact assay interpretation. Together, these protocols provide a comprehensive framework to support reproducible salivary diagnostics in diverse populations and study settings.

*NOTE*: All protocols involving human subjects must be approved and conform to governmental regulations. Appropriate informed consent is necessary for the collection and use of human study material.

## Basic Protocol 1 SALIVA COLLECTION BY THE PASSIVE DROOL METHOD

Methods for saliva collection, processing, and storage can have a large affect on results in salivary protein assays, making it critical to carefully consider and standardize these procedures when designing studies. Factors such as time of day, hormonal status (e.g., menstrual cycle phase or hormone replacement therapy), and pre‐collection activities such as sleep, exercise, diet, oral hygiene maintenance (toothbrushing and mouthwash use), and medication use introduce variability in salivary measures. These variables should be documented during collection and, when appropriate, controlled for in biomarker analyses. The impact of eating, drinking, and oral hygiene behaviors on salivary biomarkers can be mitigated by instructing subjects to avoid such activities one hour before sample collection and performing a water rinse immediately prior to saliva collection (see Supporting Information Document S1). Other factors (e.g., last time of exercise, last menstrual period) can be measured and controlled in analyses using standardized metadata collection forms (see Supporting Information Document S2).

Several approaches are available for saliva collection, each with strengths and limitations that vary depending on the analytes of interest and study design. Significant and clinically relevant differences in salivary biomarker levels have been observed across collection methods (Gallagher et al., 2006; Topkas et al., 2012), so the choice of method should be carefully considered in relation to its reproducibility for measuring the analytes of interest. Methods include collecting pooled whole saliva, facilitation of collection using absorbent material (e.g., cotton swabs), or collection using mouthwash swirls (Granger et al., 2012b). Stimulated saliva collection can also be conducted with a participant producing saliva using chewing gums or waxes, but this may affect the saliva composition compared to unstimulated conditions and is usually reserved for specific research questions (Granger et al., 2012b). Unstimulated pooled saliva collection (i.e., passive drool) offers several advantages: it is validated for most salivary biomarkers, allows visual confirmation of sample volume and calculation of flow rate, is low in cost, and yields relatively large volumes. Its main disadvantages are that it requires participant cooperation and a certain level of skill to follow the collection procedures accurately. Other collection methods may be more appropriate when study procedures require movement or the performance of activities that make passive drool saliva collection challenging. For example, cotton or synthetic swabs may be more practical when collecting saliva during a simulated or actual task or in relation to exercise where stopping to collect passive drool saliva samples would be impractical. Beyond activity‐based interventions, cotton‐based collection may be preferable in pediatric or clinical populations with low salivary flow, bedridden or mobility‐limited patients, field settings requiring scalability, or protocols involving frequent sampling, provided the analytes of interest are validated for this method (Fey et al., 2024; Møller et al., 2022).

During the collection phase, the sample should be analyzed for blood contamination, as this can interfere with measurement of inflammatory biomarkers and analytes like testosterone (Granger et al., 2012b; Kivlighan et al., 2004; Szabo & Slavish, 2021). With pregnancy and in certain populations with oral and periodontal diseases, microscopic or macroscopic blood leakage into the mouth is common. Therefore, it is recommended to check saliva for blood contamination during sample collection (Carrillo‐de‐Albornoz et al., 2012; Kang et al., 2018; Kivlighan et al., 2004). A visual inspection using a standardized scale should be carried out and is described here. This is a quick assessment and performing it during the collection phase will allow for recollection, if needed (Kang & Kho, 2019). Visual inspection may not, however, pick up trace or microscopic blood that can still influence analyte results. In this case, a transferrin assay may be preferred (see Basic Protocol 4).

The following protocol outlines the collection of unstimulated, pooled whole saliva (passive drool) along with procedures for documenting relevant clinical and behavioral patient‐specific variables that may influence salivary composition and biomarker interpretation. The passive drool method is a noninvasive method that requires a participant to sit, lean forward, and allow saliva to pool in front of their mouth. This method is acceptable and/or preferred for the analytes of interest in this article.

### Materials

Saliva collection container, at least 5‐ml volume (e.g., Sarstedt, cat. no. NC9431413)
Nonsterile straw (optional)

#### Review patient instructions (at least 1 day before collection)

1 Review participant instructions and collection procedures in advance of sample collection.
  This helps decrease any potential anxiety or uncertainty and ensures adherence to pre‐collection instructions. See Supporting Information Document S1 for an example of unstimulated saliva sample collection instructions that can be provided to study participants ahead of biospecimen collection.
2 Have participants refrain from caffeine intake during the half‐day before saliva collection and from all eating or drinking as well as tooth brushing for at least 60 min prior to collection.

#### Document relevant behaviors (just before collection)

3 Document any consumption of alcohol, caffeine, nicotine, and prescription/OTC medications prior to saliva sample collection.
  Since these factors may influence salivary biomarker characteristics, documenting the date and time of last consumption will allow researchers to adjust for their effects in statistical analyses.
4 Document date and time of previous exercise or physical activity and the presence of recent oral injury or inflammation.
  For cortisol measurement, individuals should avoid vigorous physical activity for at least 60 min prior to collection. See Supplemental Document 2 for an example of a supplemental biospecimen collection form that can be used to document relevant pre‐sample collection activities and clinical factors.

#### Collect whole‐saliva samples

5 Instruct participants to perform a water‐only rinse to remove residual food particles. After rinsing, wait at least 10 min before collecting saliva to avoid dilution of analytes.
6 Instruct participants to allow saliva to pool naturally in the mouth.
7 With their head slightly tilted forward, instruct participants to gently expel the saliva into the collection vial, either directly or using a straw.
8 Depending on the analytes of interest and study goals, instruct participants to either fill the container to a specific volume (e.g., 1 ml) marked on the container *or* collect as much saliva as possible during a specified period (e.g., 3 min). With either option, limit the total saliva collection to a maximum of 5 min.
  Sample collection times >5 min have been shown to alter saliva characteristics and impact biomarker results. In some participants, especially when dehydrated, less than the desired volume of saliva may be collected.
  In principle, each collection vial should be preweighed in order to determine the weight of saliva collected. In practice, if the same type of vial is used across samples, the weight of the vial can be standardized, as there is very little variance (see Basic Protocol 2, step 4).
9 Record the date, start time, and total duration of saliva collection to allow calculation of salivary flow rate, if needed.
  See Basic Protocol 2 for calculation.
10 Check for visible blood contamination using a visual blood contamination and discoloration scale (Table 1). Record the score.
  It is highly recommended that samples visibly contaminated with blood (score >3) be recollected, as blood will interfere with analyte measurement.

**Table 1:** Visual Blood Contamination and Discoloration Scale*^a^* *^,^* *^b^*

Rating	Description
1	Clear with no visible color
2	A hint of color; a little brown, yellow, or red/pink tint is barely visible
3	Clearly visible brown, yellow, or red/pink tint
4	Obvious brown, yellow, or red/pink color that is more than just a tint, but not very deep
5	Very apparent deep, rich, dark brown, yellow, or red/pink color

^*a*^ Different visual assessment scales can be used to evaluate for overt blood contamination in saliva samples and assist in decision making regarding the necessity of recollecting samples. This scale is adapted from Kivlighan et al. (2004).

^*b*^ Ensure that red/pink color is not from a lipstick stain.

11 Store samples on ice or at –20°C and transport to the laboratory for processing.
  If the processing laboratory is off‐site, ensure consistent handling, rapid storage at a cool temperature (up to 24 hr at or below 4°C is acceptable, although −20°C is ideal), and appropriate transport methods to maintain the cold chain (Padilla et al., 2020; Pramanik et al., 2012).

## Basic Protocol 2 PROCESSING, STORAGE, AND CHARACTERIZATION OF SALIVA (VISUAL ASSESSMENT SCALE, pH, AND FLOW RATE)

Proper processing and storage after saliva collection are essential to prevent protein degradation. This protocol outlines post‐collection handling and storage strategies designed to facilitate biospecimen management and reduce variability in future analyses.

Characteristics of saliva such as pH, flow rate, and blood contamination can interfere with assay results and be altered in certain populations and by pre‐collection conditions and collection methods. Salivary pH helps maintain oral health. An average value is ∼6.6, though it can range from 4 to 8 across individuals and depending on time of day, diet, comorbid conditions, and rate of salivary production (Dukić et al., 2013; Kubala et al., 2018; Lăzureanu et al., 2021). In addition to serving as an important clinical marker, salivary pH should be recorded for assay validation, as large fluctuations can interfere with assay binding. Samples with a pH below 3.5 or above 9.0 are generally recommended for exclusion from analysis (Schwartz et al., 1998).

Differences in salivary flow rate across subjects are common and many factors influence salivary flow, including medications, tobacco, environmental or stress‐related factors, periodontal disease, and specific conditions linked to xerostomia (Al‐Moosawi & Qasim, 2020; Beltzer et al., 2010; Dukić et al., 2013; Lăzureanu et al., 2021). Salivary flow rate can influence salivary analyte levels, depending on molecular size and degree of protein binding. For example, large blood‐borne molecules such as the steroid dehydroepiandrosterone (DHEA), as well as analytes produced by the salivary glands like alpha‐amylase and secretory IgA (sIgA), are particularly sensitive to flow rate. Recording, calculating, and statistically controlling for flow rate when analyzing these markers helps minimize variability.

Due to the viscous nature of saliva, handling saliva and preparing aliquots of salivary supernatant for analysis can be challenging. Preparing aliquots of supernatant after one freeze/thaw cycle and centrifugation to allow separation of mucins from creates a less viscous, more easily transferrable solution (Padilla et al., 2020). To minimize variability across samples, it is best to process saliva and aliquot the supernatant within a few days of the overnight freeze date (below). The flow rate is determined from the collection time and volume of the whole, unstimulated saliva sample, while prepared salivary supernatant samples are used for analyte measurements in Basic Protocols 3 and 4.

### Materials

pH meter for smaller volumes (<5 ml) and size of saliva container (e.g., Thermo Scientific, cat. no. STARA2110)
1.5‐ml microcentrifuge tubes or cryotubes
Ice or dry ice

#### Record volume and pH of saliva

1 Calibrate the pH meter according to manufacturer's instructions.
2 Thaw saliva samples completely at room temperature.
  Thawing time depends on transport and storage conditions.
3 Vortex samples to mix.
4 Weigh the container with saliva and subtract the weight of the container to obtain the total volume of saliva (1 g = 1 ml). Record the total saliva volume.
  Saliva collection containers should be weighed prior to sample collection to calculate weight‐based saliva volume. For example, if the weight of the container is 4.38 g and the total weight of the container after saliva sample collection is 9 g, then the saliva sample weight is 9 g − 4.38 g = 4.62 g. Since saliva density is 1 g/ml, the sample volume is 4.62 ml.
5 Measure and record the pH of the saliva.
  If saliva pH < 3.5 or > 9.0, the sample may yield incorrect results. If possible, recollect the sample.
6 Record the value from the visual blood contamination and discoloration scale (see Basic Protocol 1, step 10) with the other data.
7 Freeze sample overnight at –20°C or for 1 hr at –80°C.

#### Prepare salivary supernatant

8 Thaw sample at room temperature.
9 Centrifuge 15 min at 1500 × *g*.
10 Inspect to see if there is a mucin pellet at the bottom of the tube. If there is no visible pellet, centrifuge one more time.
11 Aliquot the saliva supernatant, being careful to not disturb the pellet.
  If you disturb the pellet, centrifuge the sample again and resume extracting the supernatant.
  The volume of each aliquot needed depends on the assays to be performed. Refer to Table 2 for an example based on the analytes in the following protocols.

**Table 2:** Example Aliquot Sizes

Biomarker	Recommended volume
Alpha‐amylase	75 µl
Cortisol	75 µl
Cotinine	75 µl
CRP	225 µl
sIgA	75 µl
IgG	75 µl
Transferrin	75 µl
Extra aliquots	75 µl each
Total volume	5 ml

The volume and quantity of aliquots depend on the volume of saliva collected and the assays of interest.

12 If extended preparation times (>30 min) are anticipated, it is best practice to place completed aliquot(s) on dry ice during the preparation of additional aliquots prior to long‐term storage at –80°C.

#### Calculate salivary flow rate

13 Calculate the flow rate from the duration of collection (see Basic Protocol 1) and the total volume of the whole‐saliva sample calculated in step 4 above. Record salivary flow rate with sample metadata for use in applicable biomarker analyses (see next step).
  Flow rate (ml/min) = volume collected (ml)/ time (min)
14 To use as a correction for analyte output, use the equation:
  Analyte output (µg/min) = analyte concentration (µg/ml) × flow rate (ml/min)
  Some analytes such as large blood‐borne molecules (dehydroepiandrosterone) or those secreted by the salivary glands (alpha‐amylase, sIgA) are flow‐rate dependent and require correction by calculation of analyte output.

## Basic Protocol 3 QUANTIFICATION OF COTININE IN SALIVARY SUPERNATANT

This protocol describes the quantification of the patient‐specific factor cotinine. Cotinine is formed by the breakdown of nicotine and is the preferred marker of nicotine use as it has a longer half‐life (17 hr) compared to nicotine (2 hr) (Benowitz, 1983). The following steps detail procedures and results from the Salimetrics cotinine ELISA kit and may require modification if an alternate kit is used.

### Materials

Cotinine ELISA kit (Salimetrics, cat. no. 1‐2002) including:
  Assay diluent
  10× wash buffer concentrate
  Cotinine standard and controls (high, low)
  Enzyme conjugate
  Anti‐cotinine antibody
  TMB substrate solution
  Stop solution
  Assay plate with adhesive cover
Type I water

37°C microplate incubator/rotator (VWR, cat. no. 12620‐930)
Aluminum foil
1.5‐ml microcentrifuge tubes (or other small tubes)
15‐ml or larger conical tube
Precision multichannel pipette to deliver 50‐200 µl
Reagent reservoir to hold ≥15 ml
Microplate washer (*optional*; BioTek, cat. no. 405TSRVS405)
Plate reader with 450 nm and 620‐630 nm reference filters (BioTek, cat. no. 1120531)
Computer software for data reduction (BioTek GEN5 version 3.14)

*NOTE*: It is important to read the manufacturer's instructions and prepare reagents accordingly before beginning the assay.

#### Prepare reagents and samples

1 Preheat the microplate incubator/shaker to 37°C.
2 Determine the plate layout to include all standards, controls, samples, and blanks (if used) in duplicate.
  Blanks (i.e., wells containing all assay reagents except the sample or standard) should be included when it is necessary to measure background signal from the plate or reagents, or when no true zero standard is available, to confirm that measured absorbance reflects the analyte rather than nonspecific activity.
3 Bring all reagents to room temperature (18°‐25°C) prior to use. Bring microtiter plate to room temperature, kept in foil until use.
  A minimum of 1.5 hr is recommended to warm the assay diluent.
  If a precipitate has formed in the wash buffer concentrate, it may be heated to 40°C for 15 min, but should be brought to room temperature before use.
4 Prepare 1× wash buffer by diluting the 10× concentrate 10‐fold with room temperature Type I water.
  For example, add 100 ml wash buffer concentrate to 900 ml Type I water.
5 Prepare serial dilutions of the cotinine standard as follows:
  a. Label six microcentrifuge tubes 2‐6 and “zero”.
  b. Pipette 100 µl assay diluent into each tube.
  c. Serially dilute the cotinine standard 3× by adding 50 µl of 200 ng/ml standard (tube 1) to tube 2. Mix well.
  d. Change pipette tips and move 50 µl from tube 2 to tube 3. Mix well.
  e. Continue for tubes 4, 5, and 6.
    The final concentrations for tubes 1‐6 are 200 ng/ml, 66.7 ng/ml, 22.2 ng/ml, 7.4 ng/ml, 2.5 ng/ml, and 0.8 ng/ml. The zero has 0 ng/ml.
6 Thaw saliva samples completely at room temperature and vortex to mix.
7 For known smokers ONLY, dilute saliva samples 10× by adding 10 µl saliva to 90 µl assay diluent. For non‐smokers, run saliva samples undiluted.

#### Perform assay

8 Pipette 15 ml assay diluent into a disposable tube and set aside.
9 Pipette 20 µl of each standard, control, and saliva sample into the appropriate wells. Use 20 µl assay diluent as a negative control. If using blank wells, add 120 µl assay diluent.
10 Dilute the enzyme conjugate 300‐fold by adding 50 µl to the tube of assay diluent in step 8. Immediately mix and add 100 µl to each well using a multichannel pipette.
  The conjugate tube may be centrifuged for a few minutes to bring the liquid down to the bottom of the tube before taking an aliquot.
  Use a multichannel pipette for this and all subsequent reagent additions.
11 Add 100 µl cotinine antiserum to all wells except the blanks (if used).
12 Place the adhesive cover over the plate and mix in the 37°C microplate incubator/shaker continuously at 500 rpm for 1.5 hr.
  Spillage may occur if mixing speed exceeds 600 rpm.
13 Machine wash four times with 300 µl of 1× wash buffer. After each wash, invert the plate to discard the contents, then tap four or five times on absorbent paper towel to completely remove the liquid.
  Washing may be done manually by using a squirt bottle or by pipetting the wash butter into each well and discarding the liquid. After each wash, blot the plate over absorbent material.
14 Add 200 µl TMB substrate solution to each well and mix on the plate rotator for 5 min at 500 rpm.
15 Incubate in the dark (covered with foil) for 25 min at room temperature.
16 Add 50 µl stop solution to each well and mix on the plate rotator for 3 min at 500 rpm.
  If a green color remains, continue mixing until the color turns to yellow. Be sure all wells have turned yellow.
17 Read absorbance on a microplate reader at 450 nm. Read within 10 min of adding stop solution.
  A secondary filter correction at 620‐630 nm is recommended, if possible.

#### Perform calculations

**Figure 1:**
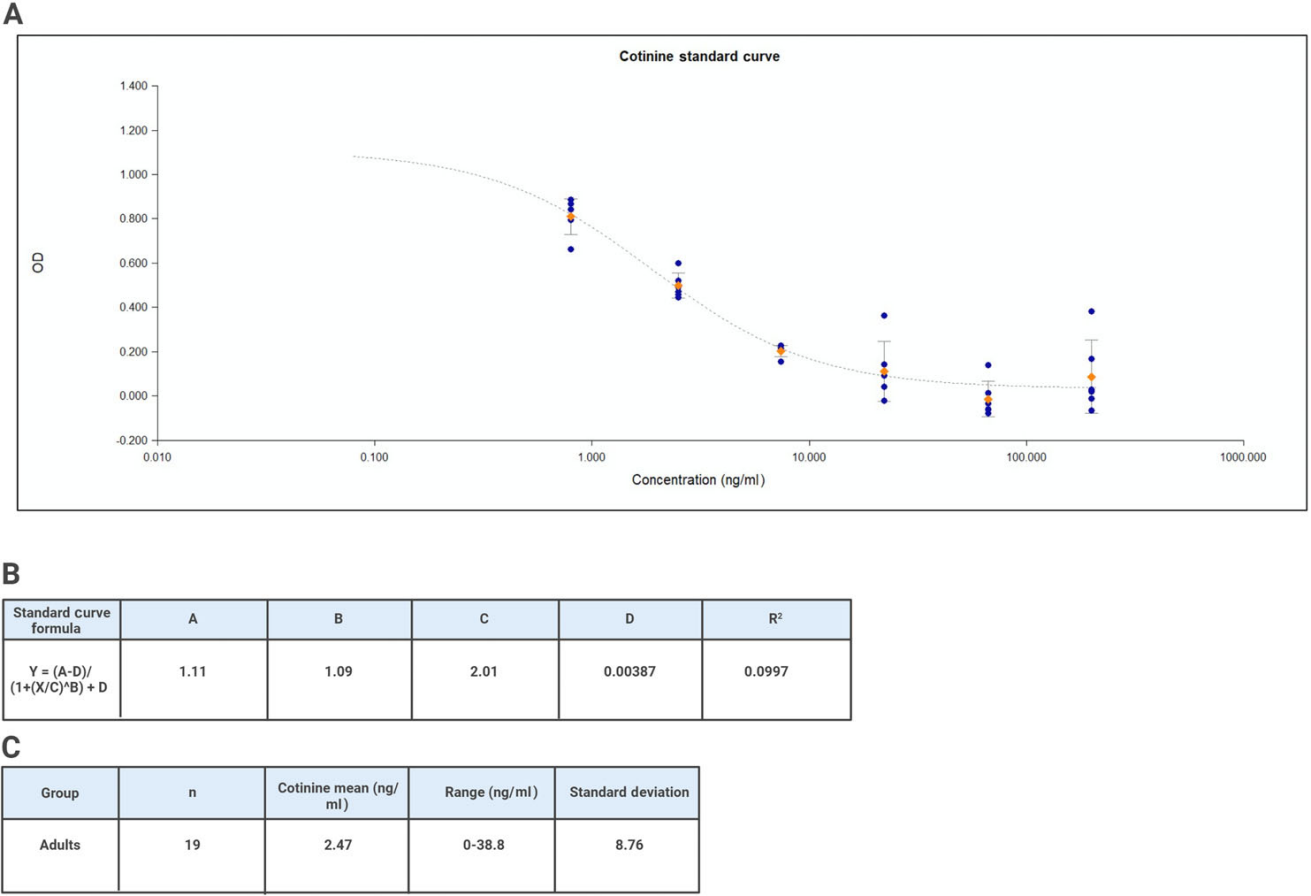
Standard curve and results generated from Salimetrics cotinine ELISA kit. (**A**) Standard curve with four‐parameter non‐linear regression curve fit. (**B**) Standard curve formula and *R* ^2^ value. (**C**) Assay results. The mean concentration of cotinine across 19 adult saliva samples was 2.47 ng/ml with a range of 0‐38.8 ng/ml. Values may differ in other populations.

18 Compute the average optical density (OD) for all duplicate wells.
19 If blank wells were used, subtract the average OD for the blanks from the OD of the zero, standards, controls, and saliva samples.
20 Determine the concentrations of the controls and saliva samples by interpolation using data reduction software. We recommend using a four‐parameter non‐linear regression curve fit.
21 If samples were diluted, multiply the assay results by the dilution factor.
  An example of a standard curve and assay results can be found in Figure 1.
  Samples with cotinine values >200 ng/ml (or >2000 ng/ml after multiplying by the dilution factor of 10) should be diluted with assay diluent and rerun for accurate results.

## Basic Protocol 4 QUANTIFICATION OF TRANSFERRIN IN SALIVARY SUPERNATANT

Transferrin is a blood glycoprotein and is a quantitative method to measure microscopic blood contamination in saliva. Measuring transferrin using an ELISA enables detection of trace blood contamination that may confound salivary analyte and immunoassay results, which cannot be reliably identified through visual inspection alone (Kang & Kho, 2019; Szabo & Slavish, 2021). The following steps detail procedures and results from the Abcam transferrin ELISA kit and may require modification if an alternate kit is used.

### Additional Materials (also see Basic Protocol 3)

Transferrin ELISA kit (Abcam, cat. no. ab108902) including:
  10× diluent N concentrate
  20× wash buffer concentrate
  Biotinylated transferrin detector antibody
  100× streptavidin‐peroxidase concentrate
  Transferrin standard
  Chromogen substrate
  Stop solution
  Assay plate coated with anti‐transferrin antibody
  Sealing tape

*NOTE*: It is important to read the manufacturer's instructions and prepare reagents accordingly before beginning the assay.

#### Prepare reagents and samples

1 Determine the plate layout to include all standards, controls, samples, and blanks (if used) in duplicate.
  If there is visible blood contamination (discoloration) in saliva samples, prepare two additional dilutions (for example, 400× and 800×) and allocate four extra wells per sample to run these dilutions in duplicate. Record the dilution factors so the results can be corrected in the data analysis (see step 23).
  Blanks (i.e., wells containing all assay reagents except the sample or standard) should be included when it is necessary to measure background signal from the plate or reagents, or when no true zero standard is available, to confirm that measured absorbance reflects the analyte rather than nonspecific activity.
2 Equilibrate all reagents to room temperature (18°‐25°C) prior to use.
3 Prepare 1× diluent N by diluting the 10× concentrate 10‐fold with Type I water. Mix gently and thoroughly.
  The 1× diluent can be stored up to 1 month at 4°C.
4 Prepare 1× wash buffer by diluting the 20× concentrate 20‐fold with Type I water. Mix gently and thoroughly.
5 Prepare 1× biotinylated transferrin antibody by diluting the 50× concentrate with 1× diluent N.
  Each well requires 50 µl of 1× antibody.
6 Spin down the 100× streptavidin‐peroxidase conjugate briefly and then prepare 1× conjugate by diluting 100‐fold with 1× diluent N.
  Each well requires 50 µl of 1× antibody. Any remaining solution should be frozen at –20°C.
7 Dilute all saliva samples 200‐fold with 1× diluent N.
  If there is visible blood contamination, prepare two extra dilutions at 400‐ and 800‐fold. *Record the dilution factor for each sample, as results will be adjusted for dilution during data analysis (see step 23)*.
8 Prepare serial dilutions of the transferrin standard.
  a. Find the mass of the transferrin standard on the vial label and calculate the appropriate volume of 1× diluent N to give a 100 ng/ml standard.
  b. Briefly spin down the contents of the vial, then add the calculated volume of diluent.
  c. Mix gently and thoroughly, then allow to sit for 10 min with gentle agitation before diluting.
  d. Label eight microcentrifuge tubes 2‐8 and “zero”.
    The reconstituted standard is tube 1.
  e. Pipette 120 µl 1× diluent N into tubes 2‐8 and 200 µl into the zero tube.
  f. Serially dilute the standard 2× by transferring 120 µl reconstituted standard (tube 1) to tube 2. Mix well.
  g. Change pipette tips and repeat for tubes 3‐8. Mix well after each transfer.
    The final concentrations for tubes 1‐8 are 100 ng/ml, 50 ng/ml, 25 ng/ml, 12.50 ng/ml, 6.25 ng/ml, 3.125 ng/ml, and 1.563 ng/ml. The zero has 0 ng/ml.
    Any remaining reconstituted standard can be used for up to 30 days when stored at –20°C. A fresh set of dilutions should be prepared immediately before for each use.

#### Perform assay

9 Remove excess microplate strips from the plate frame and return them immediately to the foil pouch with desiccant. Reseal the pouch securely to minimize exposure to water vapor.
10 Add 50 µl of all controls, standards, and samples to the appropriate wells.
11 Cover wells with sealing tape and incubate 2 hr at room temperature in the microplate incubator/shaker at 500 rpm. Start the timer after the last sample addition.
  Spillage may occur if mixing speed exceeds 600 rpm.
12 Machine wash six times with 300 µl of 1× wash buffer. After each wash, invert the plate to discard the contents, then tap four or five times on absorbent paper towel to completely remove the liquid.
  Washing may be done manually by using a squirt bottle or by pipetting the wash butter into each well and discarding the liquid. After each wash, blot the plate over absorbent material.
13 Add 50 µl of 1× biotinylated transferrin antibody to each well and incubate at room temperature and shake at 500 rpm for 1 hr.
14 Wash microplate as described above.
15 Add 50 µl of 1× SP conjugate to each well and incubate at room temperature and shake at 500 rpm for 30 min.
16 Wash microplate as described above.
17 Add 50 µl chromogen substrate per well and incubate at room temperature and shake at 500 rpm for 20 min.
18 Add 50 µl stop solution to each well.
  The color will change from blue to yellow.
19 Read absorbance on a microplate reader at 450 nm within 10 min of adding stop solution.

#### Perform calculations

**Figure 2:**
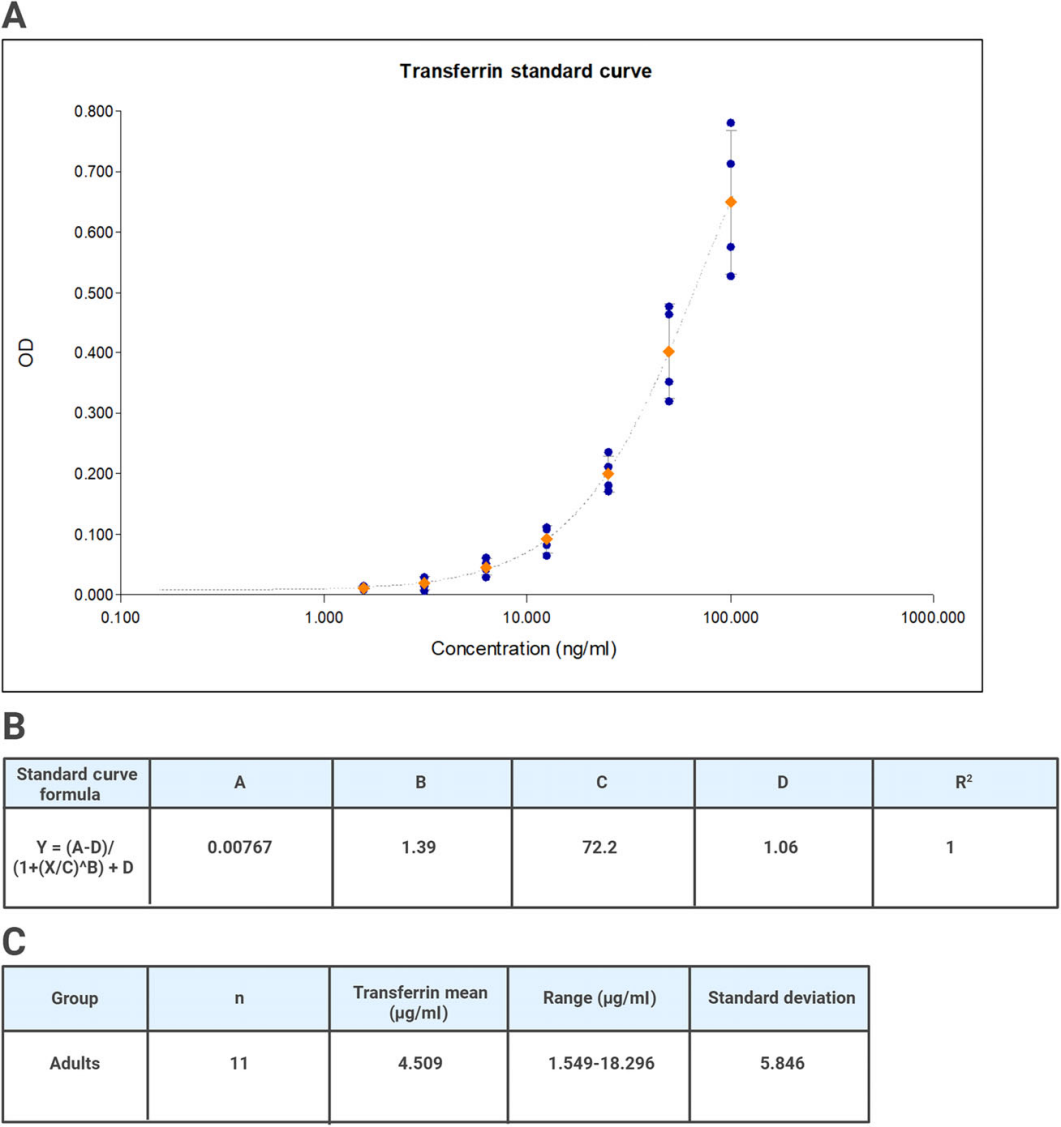
Standard curve and results generated from Abcam transferrin ELISA kit. (**A**) Standard curve with four‐parameter non‐linear regression curve fit. (**B**) Standard curve formula and *R* ^2^ value. (**C**) Assay results. The mean concentration of transferrin across 11 adult saliva samples was 4.509 µg/ml with a range of 1.549‐18.296 µg/ml. Values may differ in other populations.

20 Compute the average optical density (OD) for all duplicate wells.
21 If blank wells were used, subtract the average OD for the blanks from the OD of the zero, standards, controls, and saliva samples.
22 Determine the concentrations of the controls and saliva samples by interpolation using data reduction software. We recommend using a 4‐parameter non‐linear regression curve fit.
23 Multiply the assay results by the dilution factor.
  Because saliva samples are routinely diluted 200× for analysis (and may also be assayed at 400× or 800× when blood contamination is observed), multiply all calculated concentrations by the appropriate dilution factor to determine the actual concentration in the original sample. An example of a standard curve and assay results can be found in Figure 2.

## Commentary

### Background Information

The accessibility and noninvasive nature of saliva collection offers clear advantages over blood or tissue sampling. However, its use in biomarker research is limited by the lack of standardized approaches to collection and analysis. To address this gap, we developed detailed methodologies informed by key foundational guidance in salivary bioscience (Granger et al., 2012b; Padilla et al., 2020; Riis et al., 2021) that provide protocols for sample collection and processing, evaluation of quantitative and qualitative salivary properties, and measurement of patient‐specific modifiers (e.g., cotinine, transferrin). These approaches aim to reduce variability and strengthen the reliability of saliva as a biospecimen for translational research studies.

The adoption of systematic and consistent methods of saliva collection, processing, storage, and analysis facilitates the use of salivary biomarkers to provide insight into local and systemic physiology. Although a full discussion of the range of salivary analytes used in translational research is outside the scope of this manuscript, stress‐associated analytes such as cortisol and alpha‐amylase as well as inflammation‐associated analytes such as sIgA, IgG, and C‐reactive protein (CRP) can be reliably measured in saliva when patient‐specific and clinical factors are considered. Patients with metabolic disorders, autoimmune diseases, and chronic stress often exhibit systemic inflammation and dysregulated immune responses that are reflected in salivary cortisol and inflammatory marker profiles. Salivary cortisol and alpha‐amylase are commonly used to assess hypothalamic‐pituitary‐adrenal axis activity and autonomic nervous system activity, respectively. In contrast, salivary immune proteins differ in their sources and physiological significance. IgA is secreted locally by plasma cells in the salivary glands and serves as a first line of mucosal immune defense. IgG diffuses primarily from systemic circulation into the oral cavity and reflects systemic immune status. CRP is produced in the liver but can be detected in saliva as a marker of systemic inflammation. Salivary cytokines such as IL‐1β, IL‐6, and TNF‐α are present at lower concentrations in saliva than in blood but provide valuable insights into both local mucosal and systemic inflammatory processes when measured with sensitive assays. Together, these analytes demonstrate the potential of salivary diagnostics to provide insights into stress physiology, inflammation, and immune regulation.

### Critical Parameters

#### Timing of saliva collection

Some analytes like cortisol, alpha‐amylase, and C‐reactive protein follow a diurnal pattern, making the timing of saliva collection an essential factor to consider in study design and interpretation of results (Hoyt et al., 2021; Van Lenten & Doane, 2016). When possible, saliva should be collected at a consistent time across participants and the date of collection should be recorded to allow for evaluation and control for variability in collection. Cortisol rises sharply to its highest level shortly after awakening and decreases throughout the day. Alpha‐amylase peaks before awakening and drops throughout the day (Nater et al., 2007). To study the awakening response of cortisol and alpha‐amylase, saliva should be collected at multiple timepoints after awakening. Diurnal variability of salivary biomarker concentrations is minimized during the midday collection window, especially for salivary cytokines and chemokines (Izawa et al., 2013).

#### Whole saliva vs. supernatant

While salivary supernatant is commonly used for biomarker assays to minimize interference from cellular debris, mucins, and insoluble material, there are situations where whole saliva may be preferable. Whole saliva preserves the full biological context of the oral environment, including epithelial cells, leukocytes, and microbial components, which can provide valuable information for studies focused on host‐microbe interactions, oral disease pathology, or mucosal immune function. It also simplifies processing by eliminating centrifugation steps, making it more practical for field studies or high‐throughput collection (Pramanik et al., 2012). However, whole‐saliva samples are more viscous and subject to variability in particulate content that may reduce assay sensitivity or reproducibility for certain analytes such as mucins. This sequestering of analyte proteins may limit their detection (Iontcheva et al., 1997; Ng et al., 2007; Wozniak et al., 2002). In contrast, salivary supernatant provides a cleaner, more consistent matrix for protein and cytokine quantification, albeit at the expense of losing information contained in the cellular fraction. Overall, the choice between whole saliva and supernatant should be guided by the scientific question, the analytes of interest, and logistical considerations for sample handling.

#### Location of saliva collection

Saliva analyte concentration varies by oral fluid subtype. For instance, alpha‐amylase concentration varies across whole, submandibular, parotid, and sublingual sampling methods (Harmon et al., 2008). To minimize variability, saliva should be collected consistently from the same sites using consistent methods.

#### Correcting for flow rate

The concentration of some analytes like large blood‐borne molecules (e.g., DHEA‐S) and those released by salivary glands (e.g., alpha‐amylase, sIgA) have generally been considered to depend partially on salivary flow rate (Beltzer et al., 2010). It is important to record flow rate during saliva processing so that this correction can be performed. The equation used to calculate salivary flow rate is given in Basic Protocol 2.

#### Oral microbiome considerations

The mucins and cellular debris that are spun down from the saliva supernatant can be used for biomarker analyses, DNA extraction, and oral microbiome analysis. Although a detailed discussion of sample preservation strategies and host DNA contamination (a common issue in oral microbiome sequencing studies) is outside the scope of this manuscript, mucins may be stored at –80°C in a preservative solution such as DNA/RNA Shield added to the mucin pellet to preserve sample integrity and nucleic acid yield.

### Troubleshooting

See Table 3 for a list of problems that can arise in the analyte analyses along with possible causes and solutions.

**Table 3:** Troubleshooting Guide for Salivary Biomarker Quantification

Problem	Possible cause	Solution
Sample concentration cannot be read	Concentration outside the detection limits of the assay	Increase or decrease sample dilution as needed
Weak signal	Reagents not at room temperature	Bring reagents to room temperature before starting experiment
	Incorrect storage of reagents	Check manufacturer's instructions for storage
	Insufficient incubation times	Ensure adequate incubation according to kit instructions or increase incubation time
	Degradation of salivary proteins in samples	Ensure consistent saliva preparation and optimal storage conditions (e.g., –20° or –80°C for long‐term storage). Minimize freeze/thaws cycles.
High background/too much signal	Insufficient washing	Ensure thorough washing. After each wash, invert plate on absorbent towel to remove residual fluid.
	Contamination of wells	Change plate sealers for each incubation step (re‐using sealers may cause contamination between wells). Do not mix at high speeds (>500 rpm).
	Incorrect dilution	Ensure proper preparation/dilution of standards and samples
	Long incubation time	If using a kit, follow manufacturer's instructions for incubation time. Do not incubate longer than needed.
	Too much time between adding stop solution and reading plate	Read plate immediately after adding stop solution (within 10 min)
Poor standard curve	Improper dilution	Ensure proper preparation/dilutions of standards
	Degradation of standard	Store standard under proper conditions
High variability	Long incubation time	If using a kit, follow the manufacturer's instructions for incubation time. Do not incubate longer than needed.
	Insufficient washing	Ensure thorough washing. After each wash, invert plate on absorbent paper to remove residual fluid.
	Contamination of wells	Change plate sealers for each incubation step (re‐using sealers may cause contamination between wells). Do not mix at high speeds (>500 rpm).
	Inconsistent sample preparation or storage	Ensure consistent saliva preparation and optimal sample storage conditions (e.g., –20° or –80°C for long‐term storage). Minimize freeze/thaws cycles.

### Understanding Results

Examples of standard curve and assay results for cotinine and transferrin are shown in Figures 1 and 2. The standard curve should have a *R* ^2^ value near 1, with low coefficients of variation (CVs) between replicates. For non‐smokers, levels of cotinine are expected to be near zero.

### Time Considerations

Saliva collection from research participants will take ∼15‐20 min. Collection of qualitative and quantitative measures (flow rate, visual assessment scale, and pH) is performed during saliva collection and processing. Saliva processing times can vary depending on the freezing method used to precipitate mucins (1 hr at –20°C or overnight at –80°C) and the number of aliquots being processed. With the number of aliquots in Table 2, the processing time (from receiving samples in the laboratory to placing them in the freezer) is ~1 hr. During saliva processing, we recommend minimizing the time samples are kept at room temperature and moving them to long‐term storage at –20°C or –80°C as soon as possible. To further prevent protein degradation, we also suggest minimizing freeze‐thaw cycles by storing in aliquots.

The time needed to conduct the ELISAs depends on the methods used. The actual analysis time is ∼2.5 hr for the Salimetrics cotinine ELISA kit and ∼4 hr for the Abcam transferrin ELISA kit. Additional time is needed to bring reagents to room temperature and to calculate the standard curve and sample concentrations.

### Author Contributions

**Shirleen Xu**: Conceptualization; data curation; formal analysis; investigation; methodology; validation; writing—original draft. **Abdul Mumuni**: Methodology; supervision; validation; writing—review and editing. **Ralph Tuason**: Methodology; supervision; writing—review and editing. **Katherine Maki**: Conceptualization; data curation; funding acquisition; investigation; methodology; project administration; resources; supervision; validation; writing—review and editing.

### Conflict of Interest

The author(s) declare no potential conflicts of interest with respect to the research, authorship, and/or publication of this article.

## Supporting information

Document S1: Example of saliva collection instructions.

Document S2: Example of saliva biospecimen collection form.

## Data Availability

The data, tools, and materials (or their sources) that support this protocol are available from the corresponding author upon reasonable request.
