# Supplementary material for: Methodological Considerations in Saliva‐Based Biomarker Research: Addressing Patient‐Specific Variability in Translational Research Protocols
Source: Curr Protoc. 2025 Oct 28;5(10):e70235. doi: 10.1002/cpz1.70235 (PMC12560811; doi:10.1002/cpz1.70235)
Supplement: Supplementary file 1 — Document S1: Example of saliva collection instructions. [file CPZ1-5-0-s002.docx]

**Supplemental Information**

**Document 1.** **Example of Saliva Collection Instructions.** Steps 1-4 will generally take around 15-20 minutes.

| **Special Instructions** | Please let the study team know if you have done any of these activities in the **past hour:**   - **Drank any liquids (including water) or ate food** - **Brushed your teeth** - **Chewed gum** - **Smoked, chewed, or vaped any products (including tobacco)** | 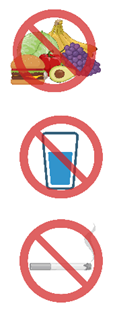 |
| --- | --- | --- |
| **Step 1** | **Rinse your mouth with water.**   - We will provide you with water to rinse your mouth to remove food residue | 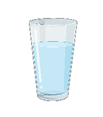 |
| **Step 2** | **Wait 10 minutes after mouth rinse.**   - After you rinse your mouth, we will need to wait a minimum of **ten minutes** before collecting your saliva sample in Step 3 | **10 Minutes**  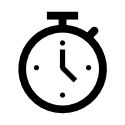 |
| **Step 3** | **Collect the sample.**   - Open the cap of the tube - Allow saliva to pool in your mouth - Then, with your head tilted forward, gently guide your saliva into the vial - If you are using a straw: place the straw in the tube opening. The straw helps guide your saliva into the tube. - Repeat this process to fill the tube to the goal volume, marked on the tube. The is the minimum amount of saliva you should aim to collect. - Collect as much saliva as you can in the 5-minute period. - If you aren’t able reach the marked line on the tube in **5 minutes**, we will use the amount of saliva you collected during the 5-minute period | **Goal Fill Line (Try to get *at least* this amount)**  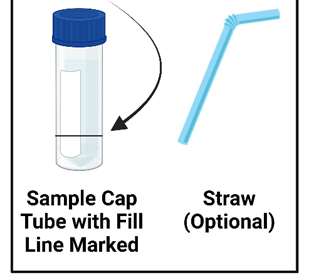  **5 Minutes**  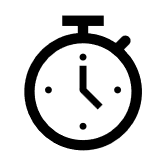 |
| **Step 4** | **Close and hand the sample to the research team**   - Attach the cap to the collection tube and tighten - Hand the saliva sample tube to the study team member |  |
| **Saliva Sample**  **Collection Complete** | **Your saliva sample collection is complete!**  Thank you! | 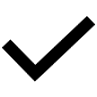 |
