# Supplementary material for: Methodological Considerations in Saliva‐Based Biomarker Research: Addressing Patient‐Specific Variability in Translational Research Protocols
Source: Curr Protoc. 2025 Oct 28;5(10):e70235. doi: 10.1002/cpz1.70235 (PMC12560811; doi:10.1002/cpz1.70235)
Supplement: Supplementary file 2 — Document S2: Example of saliva biospecimen collection form. [file CPZ1-5-0-s001.docx]

**Document 2.** **Saliva Biospecimen Collection Form**

Biospecimen Collection Form

Patient ID _____________

**Question**

- **When was the last time you had anything to eat (date/time)? _______**
- **When was the last time you had anything to drink (date/time)? ____**
- **When was the last time you consumed caffeine (date/time)? ________**
- **When was the last time you consumed dairy products (date/time)? __________**
- **When was the last time you consumed any alcoholic beverage (date/time)? ________________**
- **Sleep**
  - **When did you last go to sleep (date/time)? ______________**
  - **When did you wake up (date/time)? ____________________**
  - ***Calculated variable: {total sleep time}***
- **When was the last time you exercised (date/time)? _________________**

**Do you get menstrual periods?**

- Yes
- No
- Not Applicable

*If Yes*:

Start date of your last menstrual period: ___________

**Are you on birth control?**

- Yes
- No

**Have you begun menopause?**

- Yes
- No
- Not Applicable

**In general, how would you rate your overall health over the last 2 days?**

- Excellent
- Very Good
- Good
- Fair
- Poor
- Prefer Not to Answer
- No

**Have you had a fever within the last 7 days?**

- Yes
- No

*If Yes*:

When was the most recent date and time of your fever? ______________

**When was the last date & time you took anti-inflammatory medication(such as Advil/Ibuprofen)?**

_____________

**Have you smoked cigarettes, vaped or used tobacco products today or yesterday?**

- Yes
- No

*If Yes*:

**Product Type Used Today**

- Cigarettes
- Tobacco vaping products
- Other tobacco products

**Product Type Used Yesterday**

- Cigarettes
- Tobacco vaping products
- Other tobacco products

**Have you smoked cannabis or used cannabis vaping products today or yesterday?**

*If Yes*:

**Product Type Used Today**

- Cannabis smoked
- Cannabis vaping products

**Product Type Used Yesterday**

- Cannabis smoked
- Cannabis vaping products

**Have you consumed any alcoholic beverages today or yesterday?**

- Yes
- No

*If Yes*:

Quantity today _______

*If Yes*:

Quantity yesterday _______

**Have you consumed other non-prescribed illicit substances today or yesterday?**

- Yes
- No

*If Yes*:

Type and route ______________________

*If Yes*:

Quantity today ______________________

*If Yes*:

Quantity yesterday ______________________

**Document 2.** Saliva Biospecimen Collection Form.
